# Infant behavioral state and stool microbiome in infants receiving *Lactocaseibacillus rhamnosus* GG in formula: randomized controlled trial

**DOI:** 10.1186/s12887-022-03647-x

**Published:** 2022-10-07

**Authors:** Robert J. Shulman, Maciej Chichlowski, Fabiola Gutierrez Orozco, Cheryl L. Harris, Jennifer L. Wampler, Nicholas A. Bokulich, Carol Lynn Berseth

**Affiliations:** 1 Baylor College of Medicine, One Baylor Plaza, Houston, TX 77030 USA; 2 Center for Pediatric Abdominal Pain Research, Baylor College of Medicine, Houston, TX 77030 USA; 3 Texas Children’s Hospital, 6621 Fannin St., Houston, TX 77030 USA; 4 USDA/ARS Children’s Nutrition Research Center, 1100 Bates St., Room 8072, Houston, TX 77030 USA; 5 Medical and Scientific Affairs, Reckitt | Mead Johnson Nutrition Institute, Evansville, IN 47721 USA; 6 Laboratory of Food Systems Biotechnology, Institute of Food, Nutrition, and Health, ETH Zurich, Zurich, Switzerland

**Keywords:** Brain-gut axis, Calprotectin, Colic, Infant, Infant formula, *Lactocaseibacillus rhamnosus* GG, Microbiome, Partially hydrolyzed protein, Probiotics

## Abstract

**Background:**

Our aim was to evaluate infant behavioral state, stool microbiome profile and calprotectin in infants with infantile colic receiving a partially hydrolyzed protein formula with or without added *Lacticaseibacillus (*formerly *Lactobacillus*) *rhamnosus* GG (LGG).

**Methods:**

In this single-center, double-blind, controlled, parallel, prospective study, term infants (14–28 days of age) identified with colic (using modified Wessel’s criteria: cried and/or fussed ≥ 3 h/day for ≥ 3 days/week, in a one-week period) were randomized to receive one of two formulas over a three-week feeding period: marketed partially hydrolyzed cow’s milk-based infant formula (PHF, *n* = 35) or a similar formula with added LGG (PHF-LGG, *n* = 36). Parent-reported infant behavior was recorded at three time points (Study Days 2–4, 10–12, and 18–20). Duration (hours/day) of crying/fussing (averaged over each three-day period) was the primary outcome. Stool samples were collected at Baseline and Study End (Days 19–21) to determine stool LGG colonization (by qPCR) and microbial abundance (using 16S rRNA gene sequencing) and calprotectin (μg/g).

**Results:**

Duration of crying/fussing (mean ± SE) decreased and awake/content behavior increased over time with no significant group differences over the course of the study. There were no group differences in the percentage of infants who experienced colic by study end. Colic decreased by Study End vs Baseline in both groups. Change in fecal calprotectin also was similar between groups. Comparing Study End vs Baseline, LGG abundance was greater in the PHF-LGG group (*P* < 0.001) whereas alpha diversity was greater in the PHF group (*P* = 0.022). Beta diversity was significantly different between PHF and PHF-LGG at Study End (*P* = 0.05). By study end, relative abundance of *L. rhamnosus* was higher in the PHF-LGG vs PHF group and vs Baseline.

**Conclusions:**

In this pilot study of infants with colic, both study formulas were well tolerated. Crying/fussing decreased and awake/content behavior increased in both study groups over the course of the study. Study results demonstrate a successful introduction of the probiotic to the microbiome. The partially hydrolyzed protein formula with added LGG was associated with significant changes in the gut microbiome.

**Trial registration:**

ClinicalTrials.gov, ClinicalTrials.gov Identifier: NCT02340143. Registered 16/01/2015.

**Supplementary Information:**

The online version contains supplementary material available at 10.1186/s12887-022-03647-x.

## Background

The two-way communication between gastrointestinal and brain function is referred to as the gut-brain axis [1]. The gut microbiota play a critical role in this axis [2]. Diet plays an important role in regulating the microbial composition of the gastrointestinal tract [3, 4]. Colic, which may affect as many as 11% of infants in the first 2 months of life [5], is considered a disorder of the gut-brain axis [6]. Colic is characterized by recurrent and prolonged periods of crying, fussing, or irritability without obvious cause [6]. In addition, the abundance of *Bifidobacterium* spp. and *Lactocaseibacillus* spp. is lower in infants with colic compared with healthy infants [7–10]. Evidence supporting the use of probiotics for infantile colic is limited and still emerging. Evidence for the effectiveness of probiotics in the treatment of infantile colic was recently reviewed [11]. 

Considering gut microbiome alterations in colic, a study using next-generation sequencing identified a relative lower abundance of the genus *Lactocaseibacillus* and the phylum Firmicutes in meconium as a risk factor for the development of colic [12]. In another recent study in infants approximately two months of age fed by breast, formula, or both, colic was associated with a reduced fecal abundance of the genus *Bifidobacterium*, an increased abundance of the species *L. iners*, and *Actinobacteria spp* using next-generation sequencing [13]. In addition, infants with colic had increased fecal calprotectin, a marker of gut inflammation, compared with infants without colic, independent of the type of feeding [13]. Shannon and Simpson alpha diversity indexes did not differ between those with/without colic [13]. Pham et al., primarily using qPCR, identified *Eubacterium hallii* as being increased at 2 weeks of age in infants with colic [14]. 

Given the potential relationship between gut microbial composition and colic, studies have employed various probiotics to manage colic. One such probiotic is *Lacticaseibacillus rhamnosus* GG (LGG®; formerly, *Lactobacillus rhamnosus* GG), a non-autochthonous (i.e., transient with impermanent colonization) organism, that is a well characterized acid- and bile-stable probiotic with a long history of safe use [15–19]; preliminary evidence suggests some efficacy in the management of colic [20, 21]. Although a 2018 Cochrane Review of nutritional interventions evaluated 15 randomized controlled trials and found limited evidence for benefits associated with dietary changes for infant colic [22], dietary milk protein antigens may play a role in instigating colic. Whereas no specific expert guidelines address the elimination of cow milk and/or use of hydrolyzed protein formula to improve colic, several randomized controlled trials have reported improvement using these methods [23–28]. 

We previously studied a protein hydrolysate formula patterned after breast milk (60:40 whey-to-casein ratio, before hydrolysis) with added LGG® and demonstrated that it supported typical growth, tolerance, and safety in healthy term infants from 14-120 days of age [29]. Consequently, in the current study the primary objectives were to assess the nutritive effects of a protein hydrolysate infant formula with or without added LGG on crying/fussing and awake and alert behavior states over a 21-day feeding period using a validated parent-reported diary^14^ in infants with colic (using modified Wessel’s criteria) [30]. Introducing probiotics into the gut already populated by a large number of indigenous bacteria meets colonization resistance from the resident gut microbiota [31]. Thus, key secondary objectives were to assess stool microbiome profile, including diversity measures, and calprotectin, other tolerance measures, weight, and medically confirmed adverse events. 

## Methods

### Participants

In this single-center, double-blind, randomized, controlled, parallel-designed, prospective trial, participants were enrolled between February 2015 and June 2016 at the Children’s Nutrition Research Center at Baylor College of Medicine. The study was registered at clinicaltrials.gov (NCT 02340143). Potential participants were identified through a list of families who had expressed interest in participating in research studies performed at the Children’s Nutrition Research Center. Prior to study enrollment, mothers had chosen to primarily feed infant formula.

Participants were screened for the following inclusion criteria: term infants 14–28 days of age at the time of randomization; received ≥ 75% of the recommended caloric intake from infant formula; and whose parent answered yes to the question “Would you say that your baby cried/fussed for 3 or more hours/day within the last week?” and responded with ≥ 3 days to the question “In thinking about the last week, how many days did your baby cry and/or fuss for 3 or more hours/day?” The full list of inclusion and exclusion criteria is provided in Supplemental Table 1. The study protocol and informed consent forms were approved by the Baylor College of Medicine Institutional Review Board (Houston, Texas) and complied with good clinical practices. Mothers provided informed consent at Study Visit 1 (Baseline).

### Study design

Participants were randomly assigned to receive one of two study formulas (Mead Johnson Nutrition, Evansville, IN): a marketed partially hydrolyzed cow’s milk protein infant formula (PHF) or an investigational partially hydrolyzed cow’s milk protein infant formula with added LGG® (PHF-LGG; Chr. Hansen, Hørsholm, Denmark, 10^6^ colony-forming units per gram of powder). Both PHF and PHF-LGG study formulas were provided in a powder form.

The study sponsor created a computer-generated randomization schedule provided in opaque, consecutively numbered, sealed envelopes that was known only to the sponsor. At the study site, study formula was assigned by opening the next sequential envelope. Each study formula was designated by two unique codes (known only to the sponsor) and dispensed to parents at randomization. Product labels and sealed envelopes did not allow direct unblinding by the study site. Study monitoring personnel also were blinded to study product identification. The study sponsor could break the blind for individual participants in the event of a medical emergency in which knowledge of the study formula was critical to the participant’s management. However, it was not necessary to break the study code prematurely in this study.

At Study Visit 1 (Baseline; Day 1) eligibility was confirmed. Weight, length, and head circumference were obtained, and birth weight recorded. Parents were instructed in the use of the study formula and the study questionnaires (see below). They also were shown how to collect the stool sample for microbiome and calprotectin analysis. Parents were instructed to begin feeding study formula the next morning and continue feeding through Study Visit 2 (Day 21; end of study). On Days 2–4, 10–12, and 18–20 parents recorded formula intake over the 72-h period. Stool was collected as close as possible to study Day 1 and between Days 19–21. At Study Visit 2 (Day 21) anthropometric measurements again were obtained and study personnel recorded any adverse events and use of any medications, probiotics, or other dietary interventions taken during the study period.

### Measures

#### Baby’s Day Diary

Parents/caregivers recorded “crying/fussing” and “awake and content” infant behavior using the Baby’s Day Diary^*©*^ [32–34]. Parents completed modified daily diaries over 24-h periods with behaviors recorded in 5-min increments during three study time periods: days 2–4, 10–12, and 18–20. Parents were encouraged to fill in the diary at the same time as a repetitive activity (such as changing a diaper or feeding) or approximately every 2 to 3 h (See Supplemental Fig. 1).

#### Other tolerance outcomes

A participant was classified as having consumed breast milk if the participant consumed breast milk on any day of the three-day periods when they recorded intake. At Study Visit 2 parents completed tolerance assessments (amount of gas, average number of spit-ups per day, average number of bowel movements per day, and number of days infant cried and/or fussed ≥ 3 h/day in the week prior to the visit).

#### Stool collection

Stool samples of approximately 5 mL were collected at home and stored in the home freezer for no more than 3 days prior to couriered delivery to the study site. After delivery, samples were maintained at -80 °C until processing and analysis.

#### Stool calprotectin analysis

Stool calprotectin (μg/g) was determined using a commercially available sandwich enzyme immunoassay (PhiCal® Calprotectin ELISA, CalPro, Oslo, Norway) at a central laboratory (Cirquest, Nashville, TN). Participants taking medications prior to Study Visit 1 (Baseline) and continuing through the study were excluded from fecal calprotectin and microbiome analyses (PHF, *n* = 2). Only those participants with Baseline and Study End calprotectin and microbiome data were included in the statistical analysis.

#### Microbiome sequencing and bioinformatics

Genomic DNA was isolated and enriched and 16S rRNA gene sequencing was completed (Illumina MiSeq) as previously described (Second Genome, South San Francisco, CA) [35]. Primers used for qPCR were: F: 5’-TTGAACGCTAAAGTGACACCGTGC-3’, R: 5’-CGAAATCAGCTTGGCAAACGCCTA-3’, Probe: 5’-/56 FAM/TTGCCAGCCGAAGTTTGCATAACCGT/3IABkFQ/-3’.

Marker-gene sequence data were processed and analyzed using the plugin-based microbiome bioinformatics framework QIIME 2 version 2020.8 [36]. DADA2 [37] was used (via the q2-dada2 QIIME 2 plugin) to filter and correct sequencing errors, remove PhiX and chimeric reads, and join the paired-end sequence reads into amplicon sequence variants (ASVs). ASVs were phylogenetically placed into the SILVA version 128 reference phylogeny [38] using the q2-fragment-insertion plugin [39]. ASVs were taxonomically classified using q2-feature-classifier [40] with the classify-sklearn method against the SILVA NR99 16S rRNA reference database 138 release [38]. The SILVA reference sequences and taxonomy were formatted and filtered using RESCRIPt [41] to remove sequences with 5 or more degenerate bases, 8 or more homopolymers, sequence lengths less than 900 (Archaea) or 1200 (Bacteria), trimmed to the V4 domain using “q2-feature-classifier extract-reads” [40], and dereplicated using “rescript dereplicate” [41, 42] with default settings.

Alpha-diversity (within-sample diversity) analyses were performed using QIIME 2’s q2-diversity plugin to calculate bacterial richness (as observed sequence variants). Microbiome beta-diversity (the similarity between samples) was measured in QIIME 2 using Bray–Curtis dissimilarity, Jaccard distance, and weighted and unweighted UniFrac distance [43]. Feature tables were evenly subsampled at 78,000 sequences per sample prior to alpha and beta diversity analyses. Kruskal–Wallis tests (for non-normal data; with the q2-diversity plugin) or two-way ANOVA tests (for normally distributed data) and Wilcoxon signed-rank tests were performed (using the q2-longitudinal plugin [44] to test whether alpha diversity estimates differed between visit and feeding group. Two-way permutational multivariate analysis of variance (PERMANOVA) tests [45] (as implemented in the adonis method in the vegan R package [46], wrapped via the q2-diversity plugin) were performed to test whether beta diversity estimates partitioned by visit and feeding group.

Supervised learning was performed in q2-sample-classifier [47] to select ASVs that differentiated subjects based on study group at follow-up and predict study group via fivefold nested cross-validation (classify-samples-ncv method), using random forests classification models [48] grown with 500 trees. Taxonomic compositions in each sample (grouped by visit and feeding group) were visualized using differential heatmaps with Metacoder [49] in R [50]. Analysis of Composition of Microbiomes (ANCOM) [51] was used to test the differential abundance of microbial ASVs between feeding groups at both Baseline and follow-up.

#### Adverse events

Any adverse events or serious adverse events were collected throughout the study period and coded accordingly (e.g., otitis media) and by the body system involved.

#### Sample size

Duration of crying/fussing was the primary outcome variable. Sample size was based on a previous study that detected a group difference of 50 min of crying (standard deviation = 55 min) [52]. Using these assumptions, 30 participants per group were needed (α = 0.05, two-tailed test, power = 0.93).

#### Statistical analysis

Crying/fussing or awake and content (total daily duration of each) and daily formula intake (fluid oz/day) were averaged over each 3-day assessment period and analyzed by mixed models for repeated measures. Stool microbiome and calprotectin, other tolerance measures, and medically confirmed adverse events were secondary outcomes. Study group comparisons of the number of participants consuming breast milk were made using Fisher’s Exact test. Body weight was analyzed by ANOVA. The proportion of participants by study group who cried/fussed for ≥ 3 h/day for ≥ 3 days in the week prior to study visits; study discontinuation; and the incidence of adverse events were compared by Fisher’s Exact Test. The number of days a participant cried/fussed for ≥ 3 h/day and the amount of gas, number of spit-ups, and number of bowel movements were analyzed by the Cochran-Mantel–Haenszel row mean score test. Stool calprotectin at each time point and changes from Baseline were analyzed by the Kruskal–Wallis test.

## Results

### Study allocation and demographics

A total of 71 participants were enrolled and randomized (PHF: 35; PHF-LGG: 36; Fig. 1). Sex, race, ethnicity, birth anthropometric measures, and age at randomization were similar between groups (Table 1). Study completion rates were similar between groups (PHF: *n* = 33, 94%; PHF-LGG: *n* = 33, 92%; *P* = 1.000).

**Fig. 1:**
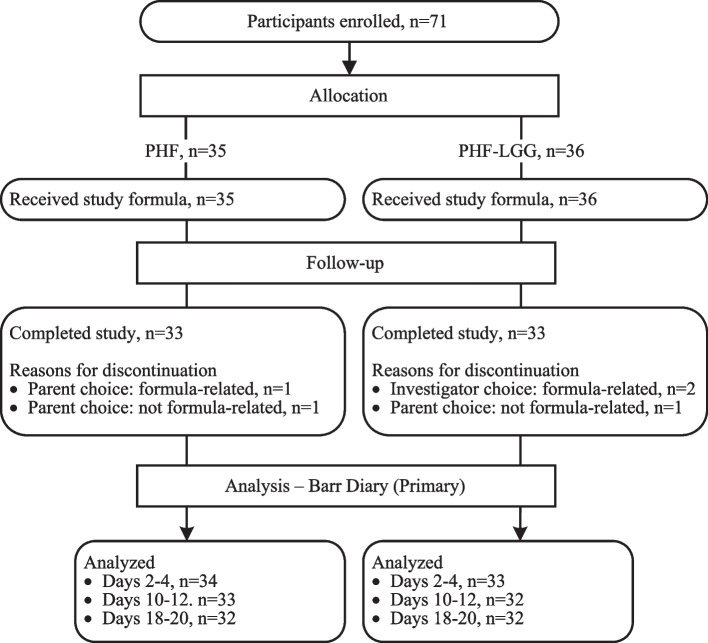
Study allocation

**Table 1:** Infant characteristics

	Study Group		*P*
	PHF, *n* = 35	PHF-LGG, *n* = 36	
Sex, n (%)			
Female	14 (40)	14 (39)	1.000
Male	21 (60)	22 (61)	
Race, n (%)			
White	11 (32)	14 (40)	0.618
Black	23 (68)	21 (60)	
Ethnicity, n (%)			
Hispanic	13 (37)	15 (42)	0.809
Non-Hispanic	22 (63)	21 (58)	
Birth anthropometrics^a^			
Weight (g)	3321.8 ± 70.9	3282.3 ± 70.1	0.690
Length (cm)	50.8 ± 0.3	50.6 ± 0.3	0.726
Head circumference (cm)	34.5 ± 0.2	34.4 ± 0.2	0.637
Age at randomization (days)^a^	19.7 ± 0.8	19.3 ± 0.8	0.693

^a^Mean ± SE

### Infant behavior and tolerance

**Fig. 2:**
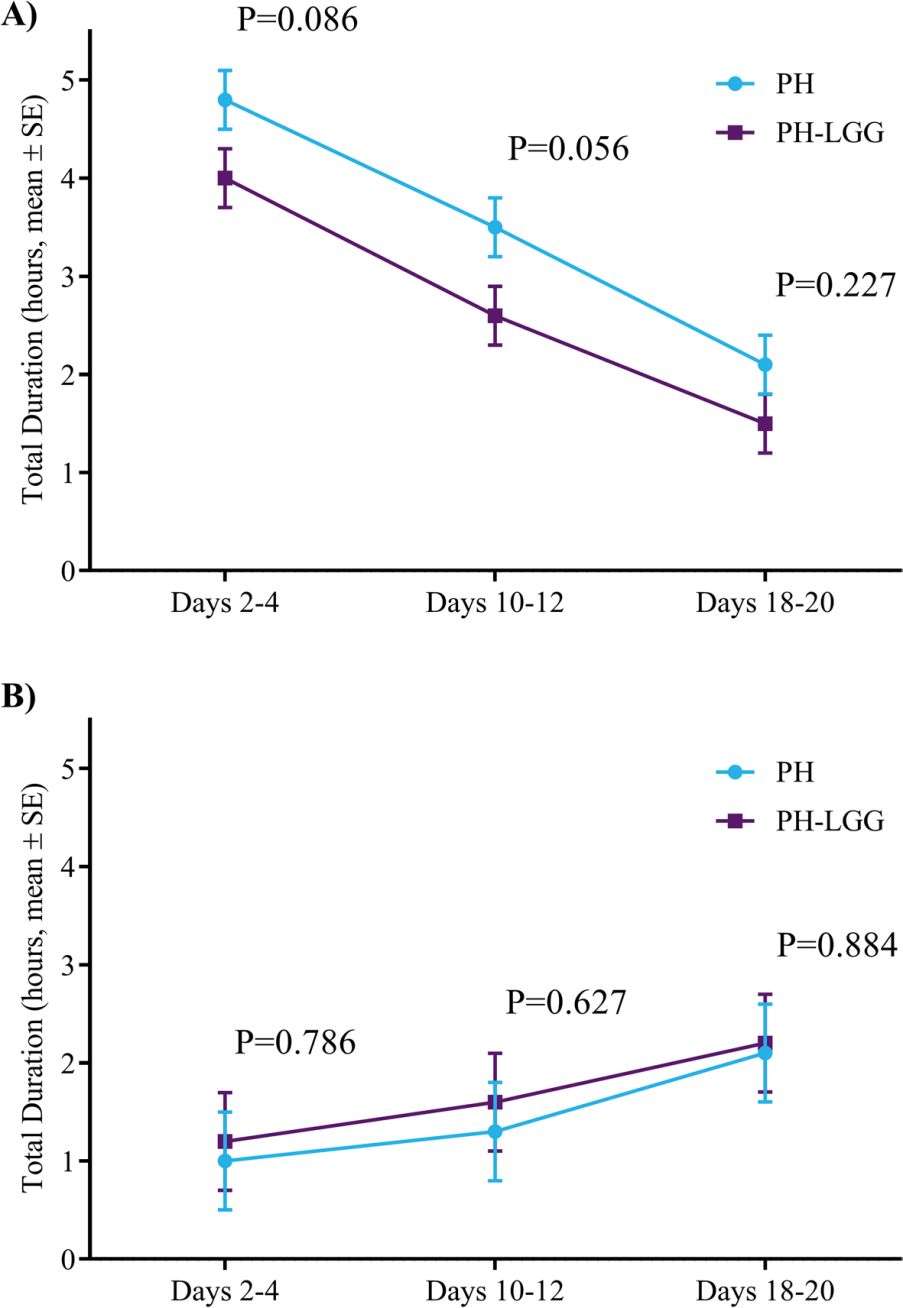
Duration (hours/day) of parent-reported **A** crying/fussing and **B** awake/content behavior. No differences were noted between groups for the change in crying/fussing or awake/content behavior. Crying/fussing decreased and awake/content behavior increased over the course of the study in both groups

A majority of parents in both groups reported that their infants (PHF: *n* = 24, 69%; PHF-LGG: *n* = 27, 75%; Supplemental Table 2) had crying/fussing for ≥ 3 h/day daily in the week prior to study enrollment. No significant difference between groups in “crying/fussing” (Fig. 2A) or in “awake and content” (Fig. 2B) duration was noted at the three study timepoints (days 2–4, 10–12, or 18–20). In both groups duration of “crying/fussing” decreased significantly (PHF: *P* < 0.001; PHF-LGG: *P* < 0.001) and duration of “awake and content” significantly increased (PHF: *P* = 0.024; PHF-LGG: *P* = 0.038) over the course of the study.

By Study End, only 8 (24%) in the PHF and 9 (27%) in the PHF-LGG group continued to cry and/or fuss ≥ 3 h/day for ≥ 3 days/week. In the week before Study End, parents reported 0 days of crying/fussing ≥ 3 h/day for over half of all participants (PHF: 17, 52%; PHF-LGG: 18, 55%). No group differences were detected in the number of spit-ups or bowel movements per day at either time point (data not shown).

### Participant characteristics and adverse events

No participants received probiotics other than LGG during the study period. There were no statistically significant differences detected in weight at Baseline or Study End (Table 1). Change in weight (g/day) during the study did not differ between groups (PHF: 39.7 ± 1.9, PHF-LGG: 40.9 ± 1.9). Study formula intake (ounces/day) was similar between groups (Table 2). Similarly, the number of infants receiving breast milk did not differ between groups (Table 2). No significant group differences were detected for study formula discontinuation either related (PHF: 0; PHF-LGG: 2, 6%) or not related to study formula (PHF: 2, 6%; PHF-LGG: 1, 3%). The number of participants who experienced medically confirmed adverse events was low (PHF: *n* = 1, 3%; PHF-LGG: *n* = 0); the participant in the PHF group reported incidents categorized as: irritability, excessive spitting, and constipation. No participant experienced a serious adverse event during the study.

**Table 2:** Study feeding intake at Days 2–4, 10–12, and 18–20

Study Period	Group	n	Study Formula, oz^a^	*P*	Breast Milk, n (%)		*P*
					Yes	No	
Days 2–4	PHF	34	24.7 ± 1.1	0.578	5 (15)	29 (85)	1.000
	PHF-LGG	33	25.6 ± 1.1		4 (12)	29 (88)	
Days 10–12	PHF	33	31.5 ± 1.1	0.776	3 (9)	30 (91)	0.613
	PHF-LGG	32	31.0 ± 1.2		1 (3)	31 (97)	
Days 18–20	PHF	33	36.7 ± 1.1	0.499	2 (6)	31 (94)	1.000
	PHF-LGG	32	35.7 ± 1.2		2 (6)	30 (94)	

^a^Mean ± SE

### LGG colonization: recovery of LGG in stool samples

**Fig. 3:**
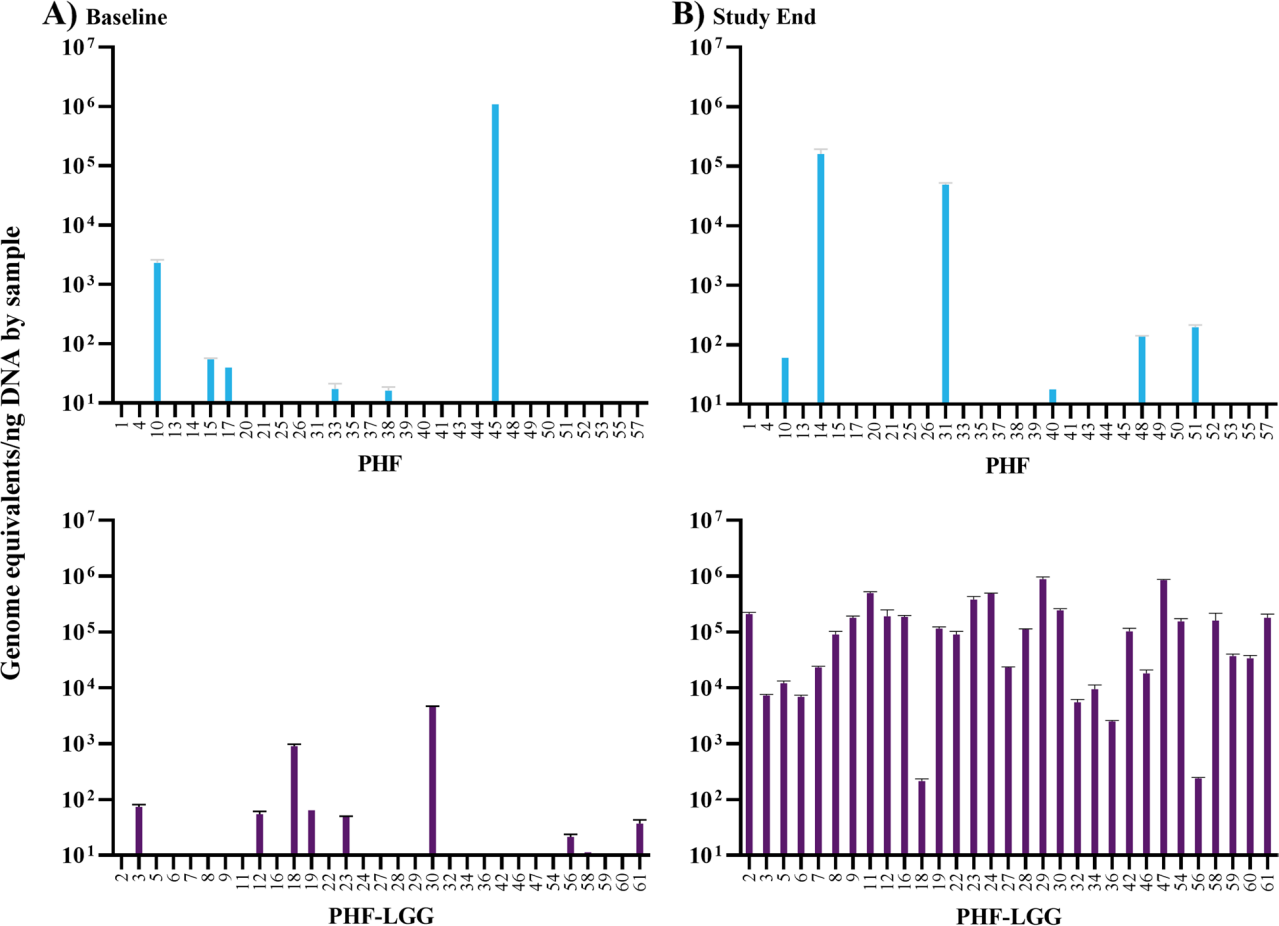
Each panel indicates genome equivalents of LGG per ng of DNA (bar height). Each group (by de-identified individual participant sample) is shown at **A)** Visit 1 (Baseline) and **B)** Visit 2 (Study End, day 21). No bar indicates that LGG was not detected. Error bars show the standard error of the mean (SEM)

No group differences in LGG quantity were detected at Baseline by qPCR (Fig. 3). At Study End, LGG quantity was significantly higher for PHF-LGG vs PHF (*P* < 0.001) and LGG was detectable in 100% of PHF-LGG vs 20% of PHF samples.

### Microbiome abundance and diversity

**Fig. 4:**
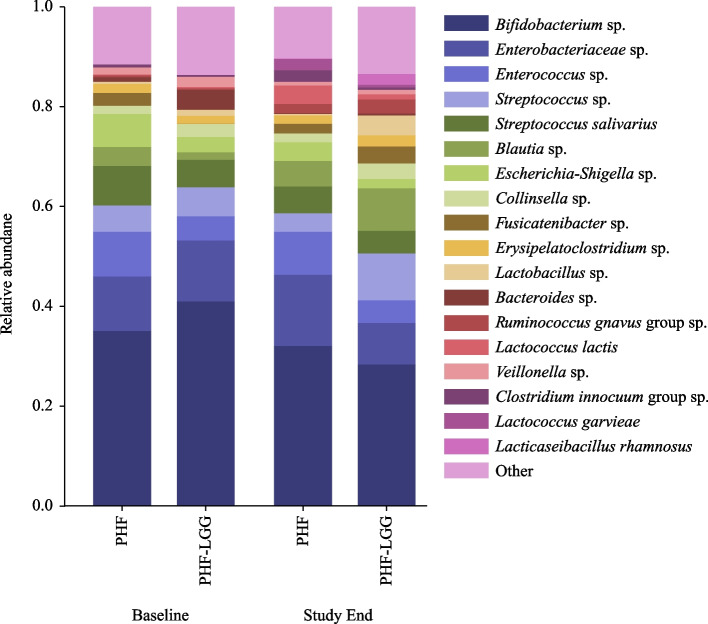
Average abundance of all species in PHF and PHF-LGG at Baseline and Study End. “Other” indicates the cumulative proportion of all taxa detected at < 0.02 relative abundance. Groups that could not be resolved at species level are labeled “sp.”. For example, “*Lactobacillus* sp.” includes all members of the genus that could be identified at genus level but excluding sequences belonging to “*L. rhamnosus*” which could be identified at species level. Hence, these labels are distinct from taxonomic classifications binned at genus level

**Fig. 5:**
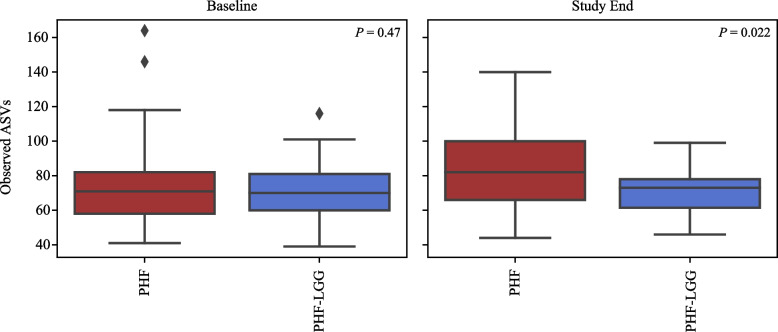
Alpha diversity (bacterial richness) measured at Baseline and Study End in all subjects. Alpha diversity was significantly lower in the PHF-LGG group at Study End (*P* = 0.022). Boxes indicate quartile measurements in each group (mid-line = median; diamonds = outliers)

**Fig. 6:**
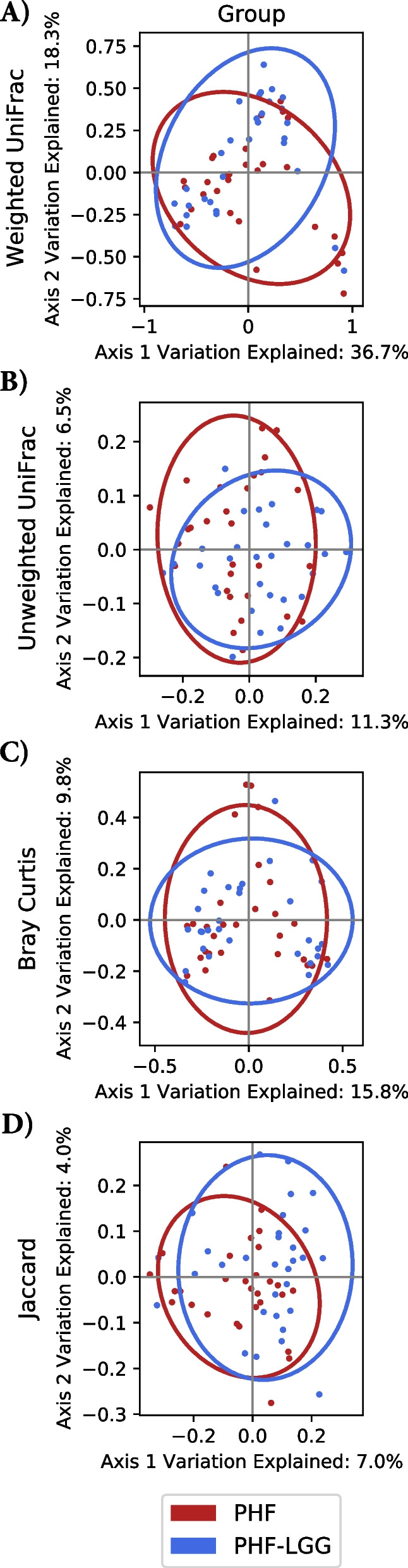
**A** Weighted UniFrac (represents phylogenetic distance or amount of branch length not shared by two samples, weighted by the abundance of each sequence variant), distances were driven by phylogenetically dissimilar, abundant groups; **B** Unweighted UniFrac, distances were driven by phylogenetically dissimilar, less abundant groups; **C** Bray Curtis (represents dissimilarities driven by differences in abundant species, regardless of phylogeny); **D** Jaccard distances (driven by differences in less abundant species, regardless of phylogeny)

Stool samples (*n* = 120) analyzed in 60 participants (PHF: *n* = 29, PHF-LGG: *n* = 31) yielded raw data composed of minimum 78,000 sequences for each sample which passed sample quality check and were used for downstream analyses. Figure 4 depicts the average abundance of all species in PHF and PHF-LGG groups at Baseline and Study End. To compare overall changes in taxonomic profiles within and between each study group, alpha and beta diversity measures were calculated. Alpha diversity was similar between groups at Baseline, but significantly lower in the PHF-LGG group at Study End (*P* = 0.022) (Fig. 5). Beta diversity measures the dissimilarity between samples, based on microbial composition. To assess community differences in beta diversity, PCoA plots were constructed for four types of beta diversity analysis. Permutational Multivariate Analysis of Variance (PERMANOVA) analysis showed no group differences at Baseline (data not shown). Groups were significantly different at Study End (i.e., both groups had significantly lower within-group beta diversity differences than between-group differences) using all distance metrics (*P* < 0.05) except for weighted UniFrac measure (Fig. 6).

**Fig. 7:**
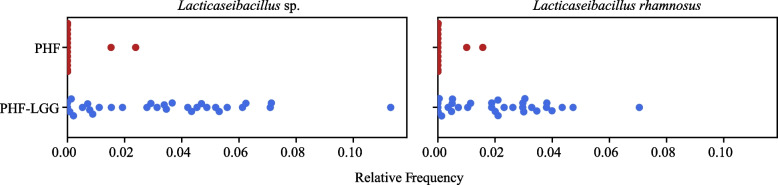
Analysis of Composition of Microbiomes (ANCOM) test used to identify species that differentiated PHF and PHF-LGG study feeding groups at Study End. The significant difference was demonstrated for two features: *Lactobacillus* spp. and *L. rhamnosus*

**Fig. 8:**
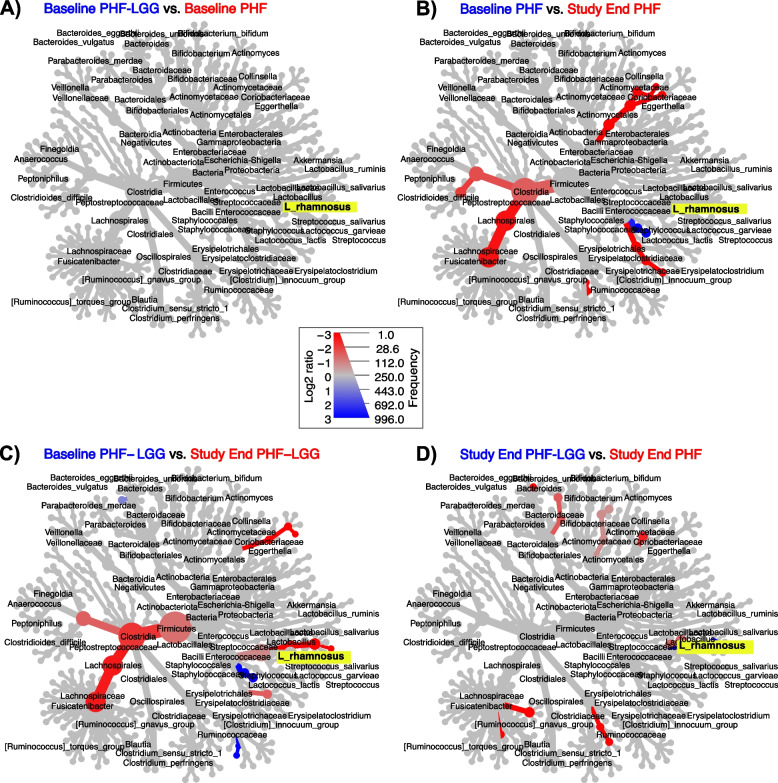
Differential heat trees based on pairwise comparisons of taxonomic relative abundance: **A** Baseline, PHF-LGG vs PHF, **B** Baseline PHF vs Study End PHF, **C** Baseline PHF-LGG vs Study End PHF-LGG, and **D** Study End, PHF-LGG vs PHF. All colored taxa are significantly different between feeding groups (Wilcoxon rank sum test; FDR *P* < 0.05) and greyed-out taxa are not significantly different. Size of nodes correspond to the number of genera and color intensity corresponds to proportions. Only taxa that were detected ≥ 10 times are displayed. *L. rhamnosus* (highlighted in yellow for easy identification) was increased in the PHF-LGG group at Study End

Analysis of Composition of Microbiomes (ANCOM) was used to identify species that differentiated study feeding groups. This analysis demonstrated a significant difference for two features: *Lactocaseibacillus* spp. and *L. rhamnosus* (Fig. 7). Random forest analysis further confirmed that abundance of *L. rhamnosus* constituted the main significant difference between PHF and PHF-LGG groups at Study End (See Supplemental Fig. 2). Relative abundance between study groups at Baseline and Study End also was exploratively compared using differential heat trees as implemented in the MetacodeR package [49] (to display taxa that are differentially abundant using a pairwise Wilcoxon test), indicating a higher relative abundance of *L. rhamnosus* detected at Study End in the PHF-LGG group than at Baseline or in the PHF group (Fig. 8).

### Stool calprotectin

Stool calprotectin was analyzed in 57 participants (PHF: *n* = 28; PHF-LGG: *n* = 29). No group difference in stool calprotectin (μg/g; median [interquartile range]) was detected at Baseline (PHF: 9.6 [4.4–28.5], PHF-LGG: 13.9 [6.8–29.2]) or Study End (PHF: 11.6 [5.6–38.7], PHF-LGG: 14.5 [5.6–24.9]). No group differences in the change of stool calprotectin from Baseline to Study End were detected (PHF: 1.7 [− 5.3–23.8], PHF-LGG: 1.0 [− 19.1–13.3]).

## Discussion

In the current randomized, double-blind trial, we enrolled a study population of infants early in life experiencing crying and fussing consistent with colic. We observed that colic improved similarly over time in infants receiving a marketed protein hydrolysate formula patterned after breast milk or a similar investigational formula with the added probiotic LGG. Infants who received the investigational protein hydrolysate formula with added probiotic demonstrated a fecal microbiome composition characterized by lower alpha diversity, significantly different beta diversity, and the presence of LGG colonization at Study End.

The seminal study that documented the natural history of crying and fussing behavior early in life recorded parent-assessed crying over the first 12 weeks of life in a cohort of non-colicky infants [53]. Stressed behavior worsened from birth until approximately 6 weeks of age, and then gradually improved, whereas in colicky infants distressed behavior peaked later and persisted through 12 weeks of age. This natural trajectory of behavior and maturation over the first 12 weeks of life was later correlated with recorded infant vocalizations and a parent-reported diary was developed and validated to help evaluate behavior patterns and potential improvement of colicky symptoms [32, 34]. Because there is a narrow time period to influence colic, the current study used a limited age window (14–28 days) for enrollment and study feeding (feeding through 5 to 7 weeks of age).

During infancy, the interaction between nutrition, intestinal microbiota, and the immune system is significant. Health benefits associated with administration of probiotics during infancy range from management of cow’s milk allergy [54] to risk reduction of immune-mediated diseases (e.g. allergy or necrotizing enterocolitis) [55]. The mechanisms of action of probiotics could be related to direct interaction of the bacteria with the host or to an indirect effect via the modulation of the resident microbiota. Many infants receive probiotic bacteria either in infant formula or as dietary supplements during their first months of life [56], however microbiota modulation by probiotics in infants is poorly understood. A key secondary outcome in the present study was evaluation of the nutritive effects of a partially hydrolyzed infant formula with added probiotics on stool microbiome. qPCR analysis was used to determine colonization by LGG and 16S rRNA sequencing was used to measure the relative abundance of dominant taxa as well as microbial richness and diversity. Using LGG-specific qPCR, we observed a robust increase in the fecal representation of this probiotic in the PHF-LGG but not the PHF group.

LGG is recognized as one of the most studied and best characterized probiotics. Thousands of infants with cow’s milk allergy have safely consumed LGG since its introduction in extensively hydrolyzed formula in 2003. Scalabrin et al. demonstrated that feeding LGG in a partially hydrolyzed formula through 120 days supported typical growth and was well tolerated by healthy infants enrolled at 14 days of age [29]. Good tolerance and colonization of LGG in a two-week feeding study was previously reported in healthy infants 0–3 months of age receiving 10^6^ cfu per gram of powdered infant formula; no differences were reported in fussiness or gas when compared to control [57]. Of note, two weeks after completing study feeding, LGG continued to be detected in a significantly higher number of participants who received the probiotic compared to the control [57]. Savino et al. showed an increase in total bacteria and *Lactocaseibacillus* and a reduction in crying time and fecal calprotectin in colicky breastfed infants supplemented with *L. rhamnosus* (5 × 10^9^ cfu/day) for 28 days, together with a maternal diet that excluded cow’s milk [20]. Administration of *L. reuteri* could contribute to management of infantile colic [58], which is associated with depressed *Lactocaseibacillus* species abundance, which precedes the onset of colic [59, 60]. Results in the current study suggest that the increase in several *Lactocaseibacillus* species is explained by the presence of the added probiotic LGG together with the stimulation of other commensal *Lactocaseibacillus* species. Results in the current study indicate a successful introduction of LGG in the gut of the infants receiving LGG in infant formula.

Whereas qPCR analysis allowed estimation of absolute LGG and other stool bacterial counts, it did not give a complete view regarding microbiota diversity. Sequencing analysis revealed multiple effects of receiving LGG including changes in alpha and beta diversity. Diversity often is considered an indicator of microbial community stability and resilience, i.e. higher diversity is associated with more stable community and lower diversity is connected with less stable community [61]. Interestingly, one exception is breastfed infants in which the microbiota has been demonstrated to be more stable over time and characterized by a lower alpha-diversity compared to infants receiving formula. Underwood et al. [62] indicated that healthy term breastfed infants are colonized by a small number of subspecies, whereas healthy term formula-fed infants are colonized by a more diverse population. Azad and colleagues demonstrated lower bacterial richness and diversity in breastfed infants and increased richness with a tendency toward higher diversity in infants receiving formula vs breastfeeding [63]. Our analysis demonstrated beta diversity between study groups was significantly different at Study End in the PHF-LGG group using all distance metrics, with the exception of weighted UniFrac. Consequently, group differences in beta diversity were significantly associated with addition of LGG in formula. A consistent diet-associated increase in family *Lactobacilliaceae* and increased *L. rhamnosus* species in infant receiving LGG in formula is highlighted in the heat tree analysis (Fig. 8).

Wide variability exists in fecal calprotectin values in both healthy infants (mean, 0–3 months of age: 145–277 µg/g) [64] and healthy children (median, 1–36 months of age: 18.1–57 µg/g) [65]. Calprotectin concentrations also have been used as a marker of inflammation [66, 67]. Dietary LGG enhances enzymatic degradation of dietary antigens [68] and decreases markers of gut inflammation in infants with atopic dermatitis and cow’s milk allergy [69, 70]. A pilot study by Fatheree et al. in colicky infants 3 to 13 weeks of age reported calprotectin concentrations in the range of 210–330 µg/g [68]. In the current study population, stool calprotectin was low at Baseline and remained low by Study End. Differences between our results and those of Fatheree et al. may relate to differences in the study populations (for example, in the previous study participating infants were older and breastfed) and/or the calprotectin assay used [68, 69]. In addition, a recent European Society of Pediatric Gastroenterology and Nutrition Gastroenterology Committee position paper reported significant variability in performance of calprotectin quantification kits exists, even from the same manufacturer [64]. Furthermore, calprotectin values in infants may also be artificially increased due to the absorption of water into the diaper or impacted by different extraction methods, for example [64]. Thus, direct comparison of calprotectin values across different studies may be difficult.

Interestingly, we observed LGG presence in 20% of the PHF group. Cross-colonization of probiotics is a common occurrence in infant clinical studies [70] and LGG is the most common probiotic used by adults (e.g. parents) [71]. Consequently, transmission via healthcare staff and the surrounding environment, respectively, have roles in this phenomenon, albeit potentially masking the true benefits of probiotic administration. Thus, potential cross-colonization in the current could account for the presence of LGG in the PHF group.

There are some limitations to our study. Parents began recording infant behavior when infants started on the study formulas. However, it is unlikely that this short period would have affected crying and fussing behavior significantly given the trajectory of parent-reported crying/fussing and awake/content behavior over the course of the study. Similarly, although some stools were collected shortly after starting study formulas, microbiome results were likely unaffected given the lack of difference between microbiome composition and diversity at Baseline compared to the striking differences demonstrated at Study End.

Strengths of the study include the double blind, randomized, controlled design; the relatively large participant enrollment; and the longitudinal design which increased the ability to detect group differences.

## Conclusions

In summary, infants receiving added LGG in a partially hydrolyzed formula exhibited significant changes in the gut microbiome composition and diversity compared to those who received formula with no added LGG. Using qPCR LGG-specific primers, we confirmed 100% colonization rate in infants receiving LGG in formula. Overall, the current study demonstrates a successful introduction of the probiotic in the gut of the infants receiving LGG in infant formula. Consumption of the probiotic LGG in a partially hydrolyzed cow’s milk-based infant formula may impact the microbial, metabolic, and immune profiles by driving beneficial downstream effects on the gut environment.

## Supplementary Information

**Additional file 1: Supplemental Table 1.** Participant inclusion and exclusion criteria. **Supplemental Table 2.** Number of days of crying/fussing for ≥3h/day in the week prior to Baseline or Study End (to meet modified Wessel’s criteria for colic).

**Additional file 2: Supplemental Figure 1. **Each 24-h period was represented by four 6-h time bars (5 min subdivisions) corresponding to: morning (6:00 a.m. to noon), afternoon (noon to 6:00 p.m.), evening (6:00 p.m. to midnight), and night (midnight to 6:00 a.m.). Crying/fussing and awake & content behaviors were recorded in 5-min increments with parents encouraged to fill in the diary every 2 to 3 hours, or at the same time as a repetitive activity, such as feeding or changing a diaper. All rights reserved Ronald G Barr, MDCM. Adapted from Barr RG, et al. 1988 Parental diary of infant cry and fuss behaviour. Arch Dis Child 63:380-387 with permission.

**Additional file 3: Supplemental Figure 2.** Random Forest classification (with 10-fold cross-validation) correctly predicts study feeding group 80% (±17.95%) on average. Panel A shows a receiver operating curve, indicating that both groups could be predicted with a high degree of accuracy vs. random chance. Panel B shows the relative importance scores for the top five most predictive sequence variants. The predictive potential was powered by a very small number of sequence variants; the top two sequence variants explain 7.4% and 6.9% of the variation, respectively, and were the same two sequence variants (*Lactocaseibacillus* spp. and *L. rhamnosus*) identified by ANCOM as being differentially abundant between the study feeding groups.

## Data Availability

The authors and study sponsor encourage and support the responsible and ethical sharing of data from clinical trials. De-identified participant data from the final research dataset used in the published manuscript may only be shared under the terms of a Data Use Agreement. Requests may be directed to: jennifer.wampler@reckitt.com. This study adheres to CONSORT guidelines.

## Abbreviations

LGG: *Lacticaseibacillus (*Formerly *Lactobacillus*) *rhamnosus* GG
PHF: Partially hydrolyzed cow’s milk-based infant formula
PHF-LGG: Partially hydrolyzed cow’s milk-based infant formula with added *Lacticaseibacillus (*formerly *Lactobacillus*) *rhamnosus* GG
