# Supplementary material for: Infant behavioral state and stool microbiome in infants receiving Lactocaseibacillus rhamnosus GG in formula: randomized controlled trial
Source: BMC Pediatr. 2022 Oct 7;22:580. doi: 10.1186/s12887-022-03647-x (PMC9541012; doi:10.1186/s12887-022-03647-x)
Supplement: Supplementary file 1 — Additional file 1: Supplemental Table 1. Participant inclusion and exclusion criteria. Supplemental Table 2. Number of days of crying/fussing for ≥3h/day in the week prior to Baseline or Study End (to meet modified Wessel’s criteria for colic). [file 12887_2022_3647_MOESM1_ESM.docx]

Supplemental TABLE 1. Participant inclusion and exclusion criteria.

| Inclusion Criteria | Exclusion Criteria |
| --- | --- |
| - 14-28 days of age at randomization (day of birth is considered day 0) - “Yes” response to Question 1 (Would you say your baby has cried/fussed for 3 or more hours/day within the last week?) on the Recall of Tolerance Questionnaire - Response of ≥3 days to Question 2 (In thinking about the last week, how many days did your baby cry and/or fuss for 3 or more hours/day?) on the Recall of Tolerance Questionnaire - Infant received ≥75% of the recommended caloric intake from infant formula over the past 24 hours - Singleton birth - Gestational age of 37-42 weeks (36 weeks and six days is considered 36 weeks gestational age; 42 weeks and six days is considered 42 weeks) - Birth weight of 2500 g (5 lbs 8 oz) or more - An English speaking/reading parent or caregiver will attend study visits - Parent or legally authorized representative agrees not to enroll infant in another interventional clinical study while participating in this study - Parent or legally authorized representative agrees not to supplement infant feedings with probiotics (including through the mother’s diet if infant receives breast milk) during study period - Signed Baylor College of Medicine IRB-approved informed consent obtained for infant’s participation in the study and, if the infant will receive some breast milk during the study, the mother will also sign consent. - Signed authorization obtained to use and/or disclose Protected Health Information for infant from birth through the length of the study period | - History of underlying metabolic or chronic disease; congenital malformation; or any other condition which, in the opinion of the Investigator, is likely to interfere with: the ability of the infant to ingest food, the normal growth and development of the infant, or the evaluation of the infant - Evidence of feeding difficulties or formula intolerance, such as vomiting or poor intake, at time of randomization (at investigator discretion) - Weight at Visit 1 is <95% of birth weight [(weight at Visit 1÷birth weight) x 100 <95%] - Infant is immunocompromised (according to a doctor’s diagnosis of immunodeficiency such as combined immunodeficiencies, DiGeorge Syndrome, Wiskott-Aldrich Syndrome, severe congenital neutropenia and secondary immunodeficiencies linked to HIV infection, Down syndrome or others) and children with known head/brain disease/injury such as microcephaly, macrocephaly or others. |

Supplemental TABLE 2: Number of days of crying/fussing for ≥3h/day in the week prior to Baseline or Study End (to meet modified Wessel’s criteria for colic)

|  | Number of Days | | | | | | | |  |
| --- | --- | --- | --- | --- | --- | --- | --- | --- | --- |
|  | 0 | 1 | 2 | 3 | 4 | 5 | 6 | 7 | P |
| Baseline, n (%)* |  |  |  |  |  |  |  |  |  |
| PHF | 0 (0) | 0 (0) | 0 (0) | 2 (6) | 3 (9) | 4 (11) | 2 (6) | 24 (69) | 0.618 |
| PHF-LGG | 0 (0) | 0 (0) | 2 (6) | 3 (8) | 3 (8) | 1 (3) | 0 (0) | 27 (75) |  |
|  |  |  |  |  |  |  |  |  |  |
| Day 28, n (%) |  |  |  |  |  |  |  |  |  |
| PHF | 17 (52) | 5 (15) | 3 (9) | 5 (15) | 1 (3) | 0 (0) | 1 (3) | 1 (3) | 0.265 |
| PHF-LGG | 18 (55) | 2 (6) | 4 (12) | 0 (0) | 3 (9) | 0 (0) | 0 (0) | 6 (18) |  |
|  |  |  |  |  |  |  |  |  |  |

* During phone screening, two parents answered “3 days” to the question: “In thinking about the last week, about how many days did your baby cry and/or fuss 3 or more hours/day?” but answered “2 days” at Study Visit 1. Data from these two participants was included in the analysis.
