# Supplementary material for: Infant behavioral state and stool microbiome in infants receiving Lactocaseibacillus rhamnosus GG in formula: randomized controlled trial
Source: BMC Pediatr. 2022 Oct 7;22:580. doi: 10.1186/s12887-022-03647-x (PMC9541012; doi:10.1186/s12887-022-03647-x)
Supplement: Supplementary file 2 — Additional file 2: Supplemental Figure 1. Each 24-h period was represented by four 6-h time bars (5 min subdivisions) corresponding to: morning (6:00 a.m. to noon), afternoon (noon to 6:00 p.m.), evening (6:00 p.m. to midnight), and night (midnight to 6:00 a.m.). Crying/fussing and awake & content behaviors were recorded in 5-min increments with parents encouraged to fill in the diary every 2 to 3 hours, or at the same time as a repetitive activity, such as feeding or changing a diaper. All rights reserved Ronald G Barr, MDCM. Adapted from Barr RG, et al. 1988 Parental diary of infant cry and fuss behaviour. Arch Dis Child 63:380-387 with permission. [file 12887_2022_3647_MOESM2_ESM.pdf]

Study Visit (Check one) : ☐ **Baseline** ☐ **Visit 2**

Recording Period (Check one): ☐ **First 24-hour period** ☐ **Second 24-hour period** ☐ **Third 24-hour period**

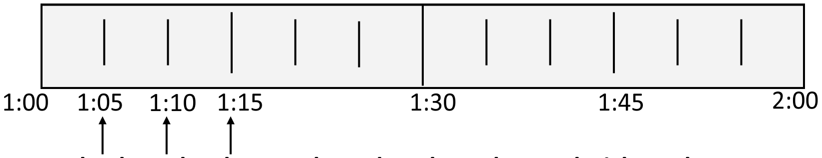

Example above has been enlarged to show that each tick mark represents a 5 minute interval

Date of First Recording on this page:

____ / ____ / ________

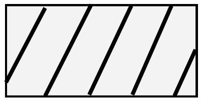

CRYING/FUSSING

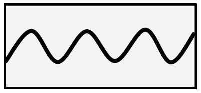

AWAKE & CONTENT

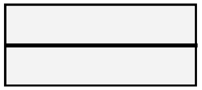

EVERYTHING ELSE (e.g., SLEEPING, EATING, CANNOT REMEMBER)

MORNING

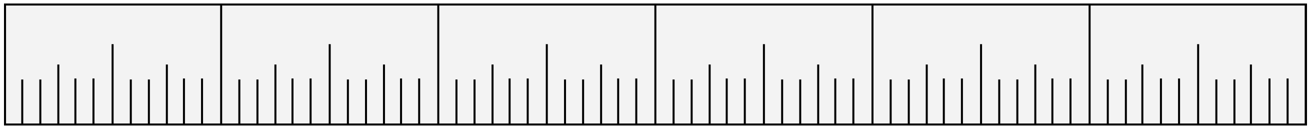

AFTERNOON

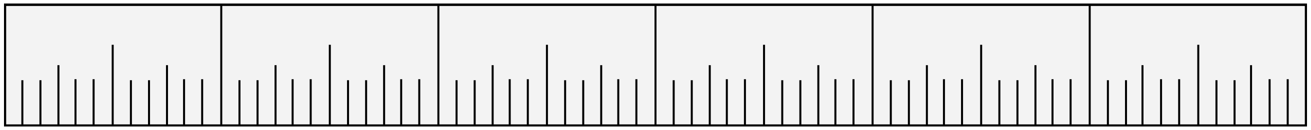

EVENING

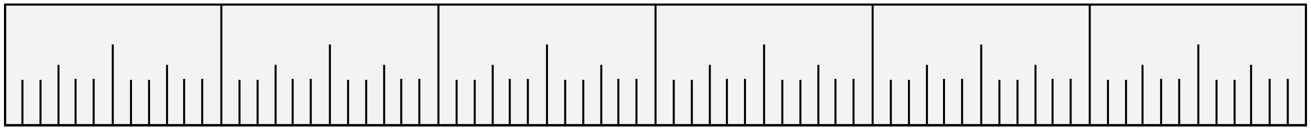

NIGHT

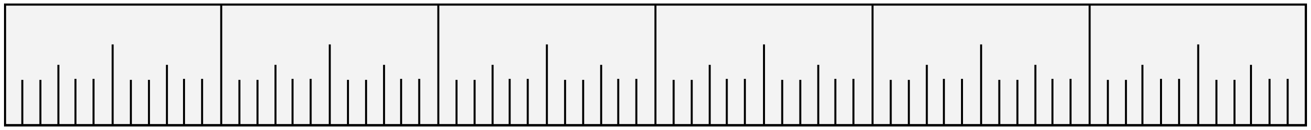

Supplemental Figure 1: Each 24-h period was represented by four 6-h time bars (5 min subdivisions) corresponding to: morning (6:00 a.m. to noon), afternoon (noon to 6:00 p.m.), evening (6:00 p.m. to midnight), and night (midnight to 6:00 a.m.). Crying/fussing and awake & content behaviors were recorded in 5-min increments with parents encouraged to fill in the diary every 2 to 3 hours, or at the same time as a repetitive activity, such as feeding or changing a diaper. All rights reserved Ronald G Barr, MDCM. Adapted from Barr RG, et al. 1988 Parental diary of infant cry and fuss behaviour. Arch Dis Child 63:380-387 with permission.
