# Supplementary material for: Infant behavioral state and stool microbiome in infants receiving Lactocaseibacillus rhamnosus GG in formula: randomized controlled trial
Source: BMC Pediatr. 2022 Oct 7;22:580. doi: 10.1186/s12887-022-03647-x (PMC9541012; doi:10.1186/s12887-022-03647-x)
Supplement: Supplementary file 3 — Additional file 3: Supplemental Figure 2. Random Forest classification (with 10-fold cross-validation) correctly predicts study feeding group 80% (±17.95%) on average. Panel A shows a receiver operating curve, indicating that both groups could be predicted with a high degree of accuracy vs. random chance. Panel B shows the relative importance scores for the top five most predictive sequence variants. The predictive potential was powered by a very small number of sequence variants; the top two sequence variants explain 7.4% and 6.9% of the variation, respectively, and were the same two sequence variants (Lactocaseibacillus spp. and L. rhamnosus) identified by ANCOM as being differentially abundant between the study feeding groups. [file 12887_2022_3647_MOESM3_ESM.docx]

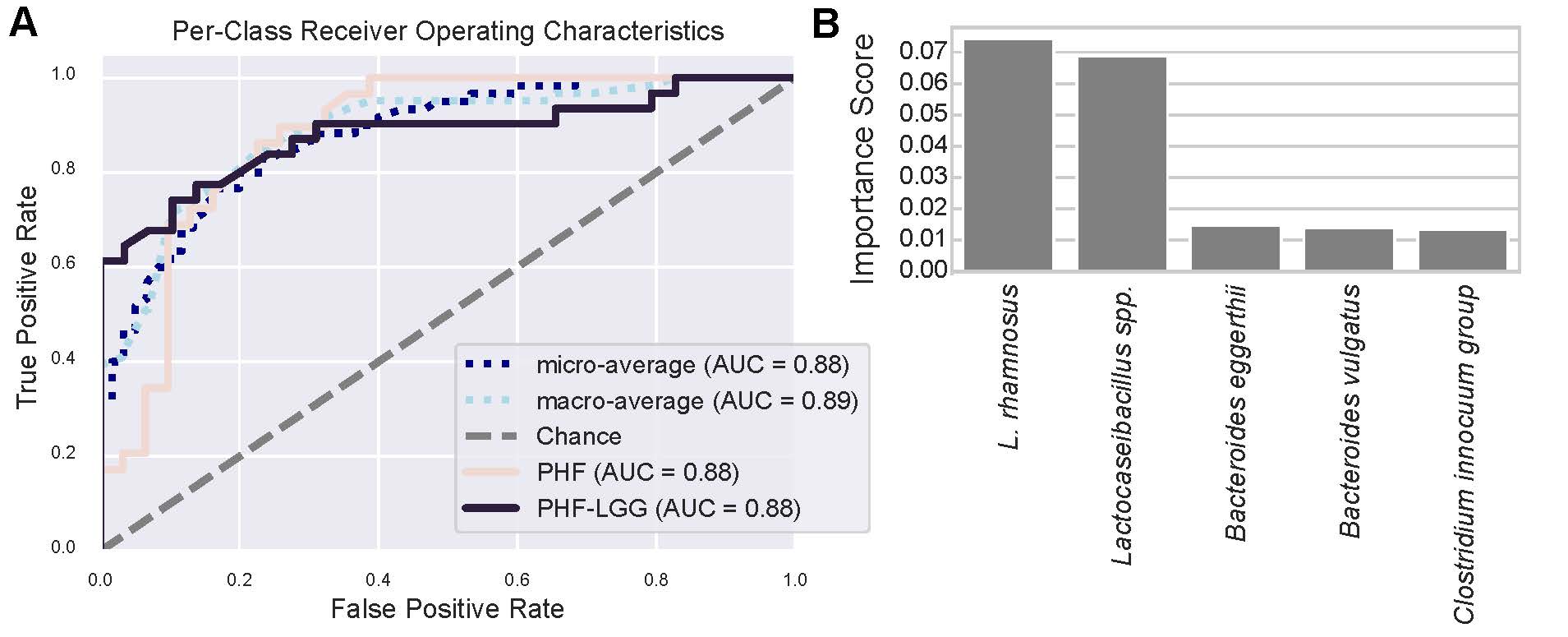


Supplemental Figure 2: Random Forest classification (with 10-fold cross-validation) correctly predicts study feeding group 80% (±17.95%) on average. Panel A shows a receiver operating curve, indicating that both groups could be predicted with a high degree of accuracy vs. random chance. Panel B shows the relative importance scores for the top five most predictive sequence variants. The predictive potential was powered by a very small number of sequence variants; the top two sequence variants explain 7.4% and 6.9% of the variation, respectively, and were the same two sequence variants (*Lactocaseibacillus* spp. and *L. rhamnosus*) identified by ANCOM as being differentially abundant between the study feeding groups.
